# Effect of stabilizers on the detection of swine influenza A virus (swIAV) in spiked oral fluids over time

**DOI:** 10.1186/s40813-024-00386-6

**Published:** 2024-11-11

**Authors:** K. Grau, K. Lillie-Jaschniski, A. Graaf-Rau, T. Harder, M. Eddicks, S. Zöls, Y. Zablotski, M. Ritzmann, J. Stadler

**Affiliations:** 1 Clinic for Swine at the Centre for Clinical Veterinary Medicine, Ludwig-Maximilians-Universität München, Oberschleißheim, Germany; 2 CEVA Tiergesundheit, Düsseldorf, Germany; 3 Institute of Diagnostic Virology, Friedrich-Loeffler-Institut, Greifswald, Insel-Riems, Germany; 4 Helmholtz Institute for One Health, Greifswald, Germany

**Keywords:** Swine, Influenza A virus, Subtyping, Surveillance, Stabilizing media, RT-qPCR

## Abstract

**Background:**

Aggregated samples such as oral fluids (OFs) display an animal friendly and time and cost-efficient sample type for swine Influenza A virus (swIAV) monitoring. However, further molecular and biological characterization of swIAV is of particular significance. The reportedly inferior suitability of aggregated samples for subtyping of swIAV presents a major drawback compared to nasal swabs, still considered the most appropriate sample type for this purpose (Garrido-Mantilla et al. BMC Vet Res 15(1):61, 2019). In addition, the viral load in the original sample, storage conditions and characteristics of different swIAV strains might further compromise the eligibility of aggregated samples for molecular detection and subtyping. Therefore, the present study aimed to evaluate the suitability of stabilizing media to minimize the degradation of viral RNA and thus increase the detection and subtyping rate of swIAV by RT-qPCR in spiked OFs under different conditions (virus strain, storage temperature and viral load in the original sample) over a time span of 14 days.

**Results:**

The use of stabilizing media in spiked OFs resulted in a significant higher probability to detect swIAV RNA compared to OFs without stabilizers (OR = 46.1, *p* < 0.001). In addition, swIAV degradation over time was significantly reduced in samples suspended with stabilizer (OR = 5.80, *p* < 0.001), in samples stored at 4 °C (OR = 2.53, *p* < 0.001) and in samples spiked with the avian derived H1N2 subtype (OR = 2.26, *p* < 0.01). No significant differences in swIAV RNA detection and degradation of swIAV RNA in spiked OFs over time were observed between the three different stabilizing media.

**Conclusion:**

Addition of stabilizers and storage of samples under cooled conditions significantly improved detection and subtyping of swIAV in spiked OFs.

## Background

Economic losses due to swine Influenza A virus (swIAV) impacting the swine industry and their potential threat to human health highlight the need for rapid and accurate identification and subtyping of these viruses [1–3].

Commonly more than one swIAV subtype circulates in one herd [4]. Frequent transmissions of human A (H1N1) 2009 pandemic virus into swine populations since 2009 established enzootically infected herds with highly variable clinical signs [5]. As a result, the already sophisticated diagnosis of swIAV is further complicated [6–8]. Whereas diagnostics for individual animals are both, time-consuming and cost-intensive, sampling techniques assessing groups of pigs have gained increasing interest concerning the detection and monitoring of various pathogens. One common aggregate non-invasive sampling method is the collection of oral fluids (OFs) [9–11], which can be collected also by staff with little previous experience. However, those specimens have shown shortcomings concerning their suitability for the subtyping of swIAV strains [9] due to lower viral loads in aggregate OF samples compared to individual samples, i.e. nasal swabs [12]. In addition, it was hypothesized that the presence of salivary enzymes [13, 14], proteins [9, 15], cellular debris [16] and high bacterial loads in oral fluids as well as inadequate transport conditions [17] can contribute to further degradation of viral RNA. To avoid degradation of intact virus or viral nucleic acids, different stabilizers have been described for the molecular detection of PRRSV [18], avian influenza virus and Newcastle Disease virus [19], SARS-CoV-2 [20], herpes simplex viruses, enteroviruses, and adenoviruses [21]. However, the stability of swIAV in aggregated samples is poorly investigated.

Therefore, the aim of the present study was to assess whether the addition of stabilizing media to OFs, spiked with two different swIAV strains, improve the rate of detection and the suitability of subtyping by RT-qPCR under different storage conditions and viral loads in the original sample over a time period of 14 days.

## Material and methods

**Fig. 1:**
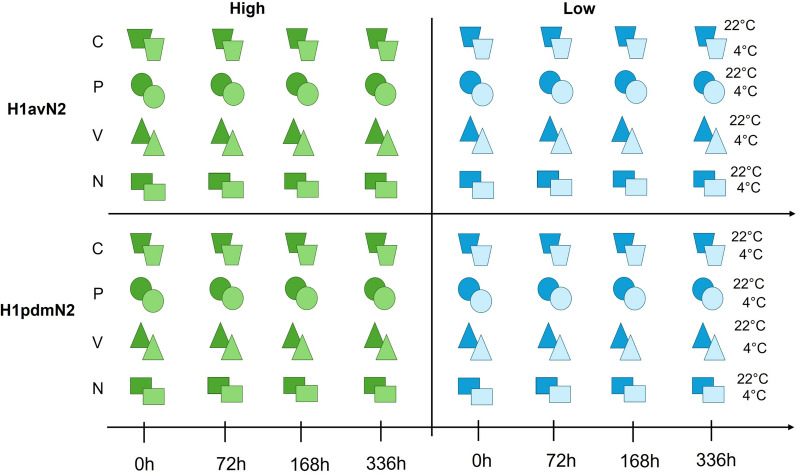
Overview of the study design. This graph shows an overall of 128 individual experiments. Oral fluids from swIAV negative pigs were spiked with H1avN2 or H1pdmN2 with either high (Ct-25) or low (Ct-32) viral loads of swIAV and suspended with stabilizing media: Virocult® (V), represented by a triangle, Primestore® (P) by a circle and NucleoProtect VET Reagent® by a rectangle. The untreated control group is represented by a trapezoid. The differentiation between darker and lighter colored shapes is intended to represent the respective storage condition (4 °C/22 °C)

OF sampling was conducted on a research facility housing SPF pigs. The facility was considered negative for swIAV based on monthly monitoring by RT-qPCR, ELISA and hemagglutination inhibition (HI) assays, all with negative results. OFs were collected individually from 24 pigs, six months of age, by using the IDEXX Oral Fluid Collection Kit (IDEXX Westbrook, USA). Briefly an undyed-cotton 3-strand twisted rope was placed into the pen at the height of the pig`s shoulder for 25–30 min, to allow the pig to chew on the rope. For extraction of the sample from the rope the wet end was inserted in the supplied plastic bag with the attached tube and manually squeezed. A total of 50 mL of OFs could be obtained from the 24 pigs. The OF was tested negative for swIAV RNA by RT-qPCR at FLI and stored at − 80 °C until further analysis. Before further investigations the samples were then thawed and centrifuged at 2000 g for 15 min. A total of 128 samples (32 samples per media (n = 96) and 32 control samples) were spiked by introducing a known quantity of a swIAV isolate into the oral fluid sample. We either used swIAV isolate A/swine/Germany/2022AI04470/2022 (H1avN2, clade 1C) or A/swine/Germany/2022AI03601/2022 (H1pdmN2, clade 1A), respectively. These virus isolates were propagated in swine testicle cells (FLI Collection of Cell Lines in Veterinary Medicine CCLV-RIE 0606) for two passages and their RNA adjusted beforehand, to achieve by RT-qPCR threshold cycle values (Ct) of 25 and 32, respectively. Afterwards, stabilizing media was added to the spiked OF specimens at a ratio of 3:1 (0.3 mL OF and 0.9 mL stabilizing media). Three different media were used (i) Sigma-Virocult® MW950S (V) (Check Diagnostics GmbH, Westerau, Germany), (ii) PrimeStore® MTM (P) (Longhorn Vaccines & Diagnostics LLC, Bethesda, USA, P) and (iii) NucleoProtect VET Reagent® (N) (MACHEREY–NAGEL GmbH & Co. KG, Dueren, Germany, M&N). Aliquots of oral fluids spiked and diluted with stabilizing media were stored at either 4 °C or at room temperature (22 °C) and triplicates of these were used for analysis at 72 h, 7 days (168 h) and 14 days (336 h). A detailed description of the study design is shown in Fig. 1.

RNA was extracted from the supernatants at the indicated time points by using 100 µL volume within the NucleoMag®VET Kit (MACHEREY-NAGEL GmbH & Co. KG, Dueren, Germany) according to the manufacturer’s instructions (MACHEREY–NAGEL GmbH & Co. KG, Dueren, Germany) and kept frozen at − 80 °C until all extractions were finalized. All RNAs were investigated in a modified generic Matrix-protein specific one step RT-qPCR for the detection of Influenza A virus [22]. Samples with a Ct-value of < 40 were rated as swIAV positive. Based on previous publications and due to lower detection limits of subtype- and lineage-specific RT-qPCRs [7, 23], samples with Ct-values of ≤ 33 were selected for subtyping as described elsewhere [24].

### Statistical analysis

The temperature, viral load, time, subtype, stabilizer (with, without) and stabilizer medium (V, P, N) were considered as potential influential factors (predictors) on swIAV positivity in a multivariable logistic regression. The backwards variable selection was used to reduce the number of predictors to only important ones. The stabilizer (with, without), viral load and time remained in the final model after backwards selection.

The temperature, viral load, time, subtype, stabilizer (with, without) and stabilizer medium (V, P, N) were considered as potential influential factors (predictors) on degradation of viral RNA in a multivariable linear regression. The backwards variable selection was used to reduce the number of predictors to only important ones. The stabilizer medium (V, P, N), subtype, temperature, and time remained in the final model after backwards selection. The normality of residuals, homogeneity of variance of residuals, linearity of residuals, the existence of influential points and collinearity of the final multivariable linear model were checked visually via the performance R-package. The assumptions were found to be satisfied.

All contrasts (differences) between categories of predictors for both logistic and linear models were assessed after model-fitting by the estimated marginal means (R package—emmeans) with Tukey *p*-value correction for multiple comparisons. Results with a *p*-value < 0.05 were considered statistically significant. Data analysis was performed using R 4.3.3 (2024-02-29).

## Results

### Laboratory investigation

#### Detection of swIAV RNA in spiked oral fluids

In general, swIAV could be detected by RT-qPCR in 109 out of 128 (85%) investigated samples. Thereof, 76 (70%) samples could be subtyped (ct-value ≤ 33). In detail 94% of the spiked samples with stabilizing media and 59% of the control samples were swIAV-RNA positive. Additionally, Ct-values ≤ 33 were found in 71% of the spiked samples with stabilizing media and in 63% of the control samples. Details on the swIAV detection and subtyping rate (Ct-values ≤ 33) in association with the different stabilizing media, viral loads, storage temperature and time after spiking are displayed in Table 1.

**Table 1:** Number (n) and percentage (%) of swIAV positive (Ct < 40) samples and samples with Ct-values (Ct ≤ 33) by influential factors viral load, swIAV subtype, stabilizer type, time after spiking and temperature

Factors	Parameter	swIAV RNA positive	Ct-values ≤ 33
Viral load	High	63/64 (98%)	53/63 (84%)
	Low	46/64 (72%)	23/46 (50%)
swIAV subtype	H1avN2	56/64 (88%)	40/56 (71%)
	H1pdmN2	53/64 (83%)	36/53 (68%)
Stabilizer	Yes	90/96 (94%)	64/90 (71%)
	No (Control)	19/32 (59%)	12/19 (63%)
	NucleoProtect VET Reagent®	29/32 (91%)	20/29 (69%)
	Primestore®	29/32 (91%)	21/29 (72%)
	Sigma-Virocult®	32/32 (100%)	23/32 (72%)
Time after spiking	0 h	32/32 (100%)	32/32 (100%)
	72 h	27/32 (84%)	17/27 (63%)
	168 h (7 d)	27/32 (84%)	15/27 (56%)
	336 h (14 d)	23/32 (72%)	12/23 (52%)
Temperature	4 °C	57/64 (89%)	42/57 (74%)
	22 °C	52/64 (81%)	34/52 (65%)

In Table 2 details on swIAV positive samples and potentially subtypeable samples with Ct-values ≤ 33 over the study period for the different stabilizing media and conditions (storage, swIAV subtypes, viral loads, temperature) are presented. Regardless of the treatment and the storage conditions all samples spiked with high viral loads (Ct-25) of H1avN2 yielded RT-qPCR positive results until the end of the study on day 14. However, only samples with stabilizers showed Ct-values ≤ 33 over the entire study period. Samples spiked at lower viral loads (Ct-32) of H1avN2 were only positive until day 14 when stored with Sigma-Virocult® and PrimeStore®. Here, cooling of Sigma-Virocult® suspended samples resulted in Ct-values ≤ 33 until day 7, whereas cooling did not affect the subtyping rate of the other samples spiked at lower viral loads (Ct-32) of H1avN2. All samples spiked with high viral loads of H1pdmN2 were positive until 14 day, when suspended with media. In contrast, only control group samples stored under refrigerated conditions yielded swIAV positive results over the entire study period. Cooling resulted in a longer possibility of subtyping for samples suspended with Sigma-Virocult®. In case of low viral loads of H1pdmN2 only samples suspended with media and stored at 4 °C were positive until day 14. Here, cooling also prolonged the period of subtyping for samples suspended with Sigma-Virocult® and Primestore®.

**Table 2:** Overview of swIAV detection by RT-qPCR in oral fluids spiked with high or low viral loads (H1avN2 or H1pdmN2) and suspended in three different stabilizer media or without stabilizer media at different storage conditions. Detected Ct-values ≥ 40 were evaluated as negative (neg.), Ct-values > 33 were not subtyped

Viral load	Temp		H1avN2				H1pdmN2			
		Medium								
		TP	C	V	P	N	C	V	P	N
High (Ct-25)	22 °C	0 h	+ ≤ 33	+ ≤ 33	+ ≤ 33	+ ≤ 33	+ ≤ 33	+ ≤ 33	+ ≤ 33	+ ≤ 33
		72 h	+ ≤ 33	+ ≤ 33	+ ≤ 33	+ ≤ 33	+ > 33	+ > 33	+ ≤ 33	+ ≤ 33
		168 h (7 d)	+ ≤ 33	+ ≤ 33	+ ≤ 33	+ ≤ 33	+ > 33	+ > 33	+ ≤ 33	+ ≤ 33
		336 (14 d)	+ > 33	+ ≤ 33	+ ≤ 33	+ ≤ 33	neg	+ > 33	+ ≤ 33	+ ≤ 33
	4°	0 h	+ ≤ 33	+ ≤ 33	+ ≤ 33	+ ≤ 33	+ ≤ 33	+ ≤ 33	+ ≤ 33	+ ≤ 33
		72 h	+ ≤ 33	+ ≤ 33	+ ≤ 33	+ ≤ 33	+ > 33	+ ≤ 33	+ ≤ 33	+ ≤ 33
		168 h (7 d)	+ ≤ 33	+ ≤ 33	+ ≤ 33	+ ≤ 33	+ > 33	+ ≤ 33	+ ≤ 33	+ ≤ 33
		336 h (14 d)	+ > 33	+ ≤ 33	+ ≤ 33	+ ≤ 33	+ > 33	+ ≤ 33	+ ≤ 33	+ ≤ 33
Low (Ct-32)	22 °C	0 h	+ ≤ 33	+ ≤ 33	+ ≤ 33	+ ≤ 33	+ ≤ 33	+ ≤ 33	+ ≤ 33	+ ≤ 33
		3 d	neg	+ > 33	+ > 33	+ > 33	neg	+ ≤ 33	neg	+ > 33
		168 h (7 d)	neg	+ > 33	+ > 33	+ > 33	neg	+ > 33	neg	+ > 33
		336 h (14 d)	neg	+ > 33	+ > 33	neg	neg	+ > 33	neg	neg
	4°	0 h	+ ≤ 33	+ ≤ 33	+ ≤ 33	+ ≤ 33	+ ≤ 33	+ ≤ 33	+ ≤ 33	+ ≤ 33
		72 h	neg	+ ≤ 33	+ > 33	+ > 33	neg	+ ≤ 33	+ ≤ 33	+ > 33
		168 h (7 d)	neg	+ ≤ 33	+ > 33	+ > 33	neg	+ ≤ 33	+ > 33	+ > 33
		336 h (14 d)	neg	+ > 33	+ > 33	neg	neg	+ ≤ 33	+ > 33	+ > 33

Ct-values ≤ 33 were considered as subtypeable. TP = time after spiking, C = control, V = Sigma-Virocult®, P = Primestore®, N = NucleoProtect VET Reagent®; neg. = negative

#### Probability to detect swIAV RNA in spiked oral fluid samples

The multivariable analysis revealed that the use of stabilizers in the spiked OFs resulted in a significant higher probability to detect swIAV RNA compared to samples without stabilizers (OR = 46.1, *p* < 0.001). In addition, the chance to detect swIAV RNA was significantly lower when OF were spiked at low viral loads (OR = 0.01, *p* < 0.001). Also, long time of storage (336 h, 14 d) reduced the probability to detect swIAV compared to immediate analysis (0 h) (OR = 0.01, *p* = 0.002) (Table 3).

**Table 3:** Odds Ratio (OR) with 95% Confidence Interval (CI) and *p*-value for the detection of swIAV RNA (dependent variable) in spiked OFs in dependency of the independent variables stabilizer, time, and viral load estimated by the multivariable logistic regression

Independent variables	Multivariable analysis		
	OR	95% CI	*p*-value
*Viral load*			
Low/High	0.01	0.00, 0.11	** < 0.001**
*Time after spiking*			
72 h/0 h	0.07	0.00, 1.34	0.094
168 h/0 h	0.07	0.00, 1.34	0.094
72/168 h	1.00	0.07, 13.6	> 0.999
336 h/0 h	0.01	0.00, 0.30	**0.002**
336 h/72 h	0.21	0.02, 2.26	0.330
336 h/168 h	0.21	0.02, 2.26	0.330
*Stabilizer*			
Without/With	0.02	0.00, 0.12	** < 0.001**

Bold values represent significant results

#### Degradation of swIAV RNA over time

**Fig. 2:**
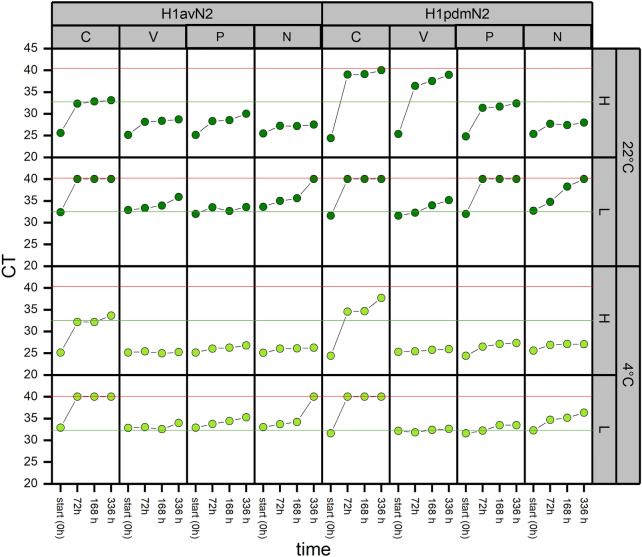
Stability of swIAV RNA in spiked oral fluids. Oral fluids from swIAV negative pigs were spiked with H1avN2 or H1pdmN2 with either high (H; Ct-25) or low (L; Ct-32) viral loads of swIAV and stored at different temperatures (room temperature, (22 °C,) or refrigerated (4 °C), with the addition of stabilizers (V = Sigma-Virocult®, P = Primestore®, N = NucleoProtect VET Reagent®) or without (C = Control). Samples were collected at predetermined intervals (start (0), 72 h, 168 h (7 d), and 336 h (14 d) after spiking) and analyzed for swIAV by RT-qPCR. The red line indicates the limit for positive samples (CT < 40) and the green line for subtypeable samples

The degradation of swIAV RNA over time was calculated by subtracting the Ct-values measured at the beginning (0 h) of the study from the respective subsequently measured Ct-values (Ct0h-72 h, Ct0h-168 h, Ct0h-336 h). The results represented as increase in Ct-values, are depicted in Fig. 2 for each stabilizer and control group samples with respect to different storage conditions and the viral loads.

**Fig. 3:**
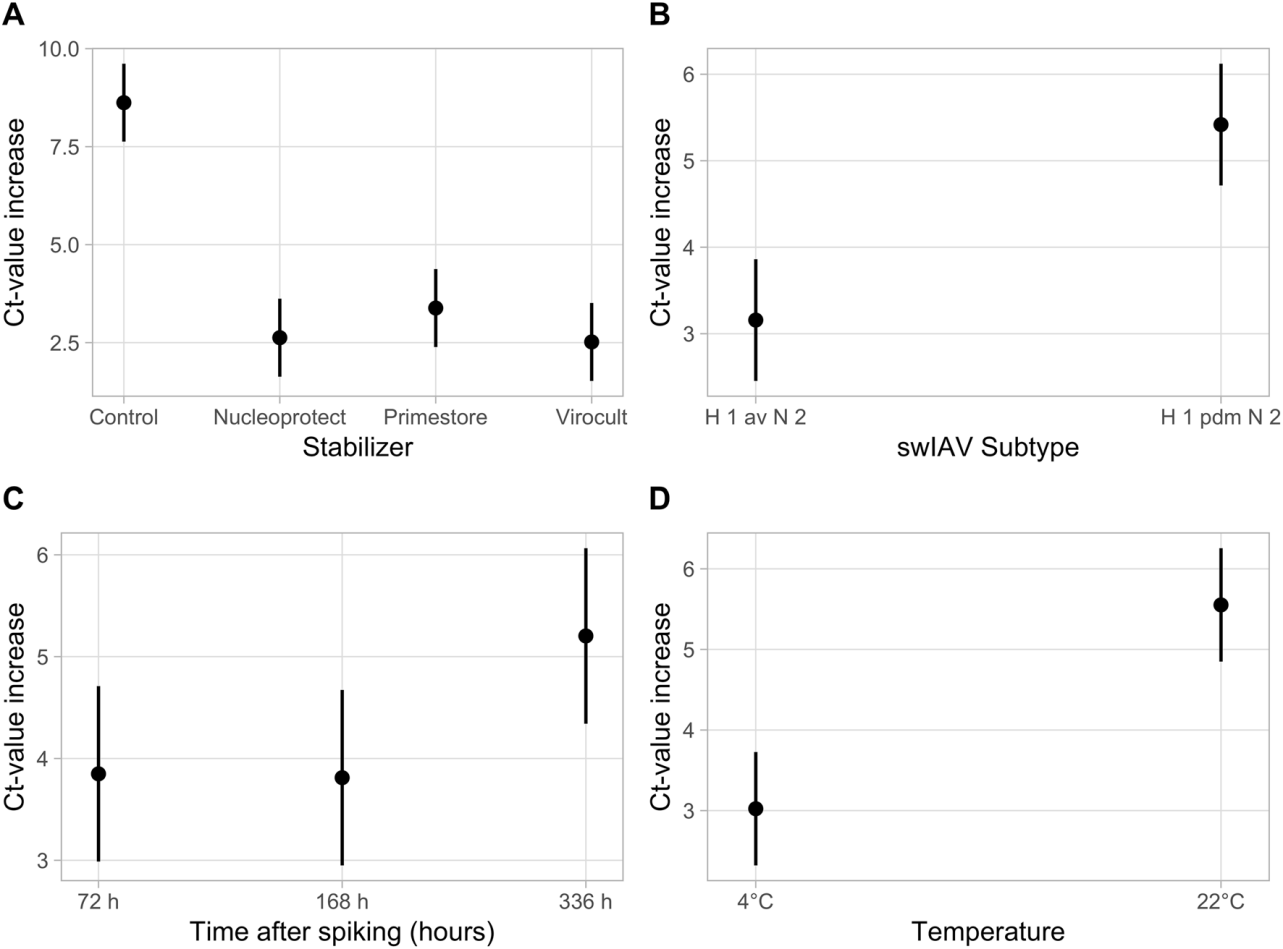
The results of the final multivariable linear regression after backwards variable selection showing the degradation of swIAV RNA, depicted as Ct-value Increase by qRT-PCR in spiked oral fluids with the addition of the different stabilizer media or without (**A**), with either H1avN2 or H1pdmN2 (**B**), collected at different time points (start (0), 72 h, 168 h (7 d), and 336 h (14 d) after spiking) (**C**) and stored at either room temperature, (22 °C,) or refrigerated (4 °C) (**D**)

The degradation of swIAV RNA over time was subsequently measured through a multivariable analysis. Adding Sigma-Virocult®, PrimeStore® or NucleoProtect VET Reagent® to OF samples resulted in a significant lower increase of the Ct-value (*p* < 0.001) compared to samples without medium. In addition, degradation of swIAV RNA over time was significantly lower in samples spiked with H1avN2 compared to samples spiked with H1pdmN2 (*p* < 0.001) and samples kept refrigerated at 4 °C compared to samples stored at 22 °C (*p* < 0.001), respectively (Table 4). The degradation of swIAV RNA, depicted as increase of Ct-values, is shown in Fig. 3

**Table 4:** Slopes (Beta), Confidence Intervals (CIs) and *p*-value of the factors (swIAV subtype, stabilizers, time after spiking, and temperature) influencing Ct-values usinga multivariable linear analysis (N = NucleoProtect VET Reagent®, C = Control, P = Primestore®, V = Sigma-Virocult®)

Independent variable	Multivariable analysis		
	Beta	95% CI	*p*-value
*swIAV subtype*			
H1avN2–H1pdmN2	2.26	1.27, 3.25	** < 0.001**
*Stabilizers*			
C–N	− 5.98	− 8.27, − 3.69	** < 0.001**
C–P	− 5.22	− 7.51, − 2.94	** < 0.001**
N–P	0.76	− 1.53, 3.05	0.8
C–V	− 6.09	− 8.38, − 3.80	** < 0.001**
N–V	− 0.11	− 2.40, 2.18	> 0.9
P–V	− 0.86	− 3.15, 1.43	0.8
*Time after spiking*			
72–168 h	− 0.04	− 1.50, 1.42	0.998
72–336 h	1.35	− 0.10, 2.81	0.074
168–336 h	1.39	− 0.07, 2.85	0.065
*Temperature*			
4–22 °C	2.53	1.54, 3.52	** < 0.001**

Bold values represent significant results

## Discussion

Since swIAV is enzootically circulating in pig populations and new subtypes with prepandemic potential might evolve, surveillance of swIAV in pig herds is of considerable importance [25, 26]. However, active surveillance of individual animals using nasal swabs is hampered by (i) the short infectious period enabling virus detection only for a couple of days and (ii) the high costs. Particularly, herds with low prevalence of swIAV require a high sample size [27]. Therefore, aggregate samples as OFs, USW or environmental samples have gained increasing interest as cost-effective and non-invasive animal friendly sampling procedures [28, 29]. However, deterioration of virus and viral RNA resulting from degradative bacterial and salivary enzymes and inhibitors [15] such as Glycoprotein-340 and MUC5B [9] might resemble a major drawback of aggregate samples like OFs and USW [30, 31], as low viral loads reduce the probability to characterize swIAV subtypes by multiplex RT-qPCR or sequencing [23, 24]. However, the identification of different strains circulating in a pig herd is of particular importance to implement prophylactic measures like vaccination as cross protection between different swIAV strains is limited [32–36]. Therefore, the effect of different stabilizers on improving the detection and subtyping rate of aggregate samples was assessed under laboratory conditions as samples with controlled laboratory swIAV contamination may not reflect samples from naturally infected pigs. Due to suitability of spiking, OFs were used for the laboratory study. Currently, three main swIAV subtypes (H1N1, H1N2, and H3N2) with a large abundance of genotypes and variants, that differ genetically and antigenically between regions, are circulating in the European pig population [8, 26, 37]. In our experiment, the subtypes H1avN2 and H1pdmN2 (clade 1C and 1A, respectively) were selected. The H1avN2 subtype, which emerged 2003 in Denmark is now predominantly found, in Denmark [8, 38], and has also been detected in e.g. France [39], Sweden, Germany [26], Spain [40], Italy [41], Belgium and the Netherlands [37]. Additionally, a pandemic strain was chosen, due to the increasing detection of pandemic strains or reassortants containing internal genes of human pandemic origin in several European countries [5, 25, 26, 37]. Interestingly, in our study degradation of swIAV RNA over 14 days of storage was significantly higher for H1pdmN2 compared to H1avN2, indicating that the real prevalence of pandemic strains is often underestimated. This finding highlights the importance of further improving shipment and storage conditions for adequate swIAV diagnostics.

The results of the laboratory study clearly show that cooled storage (4 °C) of OFs even without a stabilizer medium facilitated swIAV RNA detection up to 14 days, at least at high viral loads. According to Henao-Diaz, Giménez-Lirola [42] the most important aspects in handling of OFs includes immediate cooling of the samples (4 °C) after collection and during storage. Similar recommendations can be obtained from various authors on the management of OFs for the detection of porcine reproductive respiratory syndrome virus (PRRSV) [42–44]. However, it should be noted that in case of low viral loads, as often detected in enzootically infected farms, even at 4 °C the degradation of RNA is high and the detection rate is highly reduced. However, by adding stabilizing media and simultaneous cooling of the samples, swIAV RNA can still be detected even at low viral loads for at least 14 days. According to multivariable analysis the addition of a stabilizer did not only enable prolonged detectability of swIAV by PCR, but also increases the probability to subtype swIAV.Also, for PRRSV, another RNA virus, Decorté [45] highlighted the positive effect of stabilizer medium on the duration of PRRSV detection in oral fluids. However, Oragene RNA stabilizer (Aware Messenger, Saliva Gene Collection Module) was the only one out of three stabilizers which had a positive impact on detection of PRRSV at room temperature after 168 h [45]. In addition, other studies showed no significant impact through the addition of GTP or an antimicrobial treatment of oral fluid samples on the detection of PRRSV by RT-qPCR [43, 44].

In our experiments the performance of three different stabilizers was compared. It should be noted that molecular transport media or virus transport media have different characteristics. Whereas molecular transport media inactivates all germs in the collected sample and thus, is not appropriate for cultivation of viruses, but most suitable for the transport of viruses with a high risk of infection as e.g. such as SARS-CoV-2 [46], virus transport media still allows further diagnostics based on virus propagation [17]. In the present study, PrimeStore® and NucleoProtect VET Reagent®, were used as example of molecular transport medium. Primestore®, contains guanidine thiocyanate which inactivates germs, but still enables the detection of nucleic acids. According to the manufacturer’s instructions, the adding of NucleoProtect Vet Reagent® enables the detection of RNA for one week at RT and up to one month at 4 °C. However, in the present study OFs supplemented with NucleoProtect VET Reagent® revealed swIAV negative PCR results as soon as seven days after spiking with low viral for subtype H1avN2 stored at 4 °C. Sigma-Virocult® is a virus transport media which does not inactivate viruses. Consequently, it could be used to enable further diagnostic such as virus isolation in cell culture [17]. According to Rudsdale [47], detection of influenza A virus in samples suspended with Sigma-Virocult® is possible up to at least 8 days at both room temperature and under cooled conditions. In the present study, swIAV RNA was detectable in samples spiked with Sigma-Virocult® after 14 days (22 °C) regardless of the subtype. Based on the conditions of our study no statistically significant differences regarding both detection of swIAV and swIAV degradation over time between the three investigated stabilising media were detected. Finally, the limitations of our study (i.e. low sample size, no repetition of the experiment, limited generalizability) should be highlighted. Nevertheless, the authors believe that this exploratory study has provided important new insights in optimization of storage conditions for OFs that should be further elaborated under field conditions and for different pathogens.

## Conclusion

Addition of stabilizers improved both, detection and subtyping of swIAV in spiked oral fluids during 14 days of storage. However, it is evident that even with the addition of stabilizing media, molecular swIAV diagnostics can further be improved by refrigerated shipping and storage of the samples. Under the conditions of our study no significant differences between the three tested media were found.

## Data Availability

Data as presented in the manuskript.

## Abbreviations

av: Avian
Ct: Cycle threshold
FLI: Friedrich-Loeffler-Institute
HA: Hemagglutinin inhibition
LMU: Ludwig-Maximilians-Universität
OF: Oral fluid(s)
RT-qPCR: Real-time quantitative PCR
swIAV: Swine influenza A virus
SARS-CoV-2: Severe acute respiratory syndrome coronavirus type 2
V: Sigma-Virocult®
P: Primestore®
N: NucleoProtect Vet Reagent®
C: Control
GTP: Guanosin-5-triphosphate
